# Firwein

**Published:** 1876-07

**Authors:** A. K. Alley

**Affiliations:** Atlanta, Ga.


### FIRWEIN.

Cases Treated by Dr. A. K. ALLEY, of Atlanta, Ga.

Having read in the several medical journals notices of this new compound, *Firwein*, prepared by Messrs. Tilden & Co., of New York, so highly recommended as a new remedy, and used by some physicians with great success in pulmonary complaints, I was induced to experiment and give it a thorough test in several cases, and beg to call the attention of the profession to its beneficial and satisfactory results. I would like the profession to give it a trial, as I have found it to be a most valuable compound. It is said to contain phosphorus, iodine and bromine. I experimented with it in the three following cases with, I think, marked and decided benefit:

### CASE I.

Mrs. A., who had contracted broncho-pneumonia, which was quite prevalent in the city winter before last, and which left her right lung in a weak condition, with troublesome cough, difficulty of expectoration, and tendency to night sweats. After using all the remedies indicated in her condition, I found but little relief gained, and, seeing an article written by a physician in which "Firwein" proved signally successful, I resolved to give it a fair trial. I placed the lady upon it, and found, after using it for several weeks, that her cough was relieved, expectoration diminished, night sweats controlled, and condition of lung most decidedly improved—and her general coudition much better.

CASE II.

Mr. C., who contracted bronchitis some eight or nine years ago, was left with a troublesome cough and profuse night sweats, with general debility, and was treated by a number of physicians of eminent skill, with but little benefit. This gentleman consulted me in reference to his condition. In view of the decided improvement in the first case, from the use of "Firwein," I concluded I would try the remedy in his case, and was informed by him that nothing had given him such relief before; that his cough was under control; he had no night sweats, and that he had improved much in flesh, and was rapidly gaining his health.

CASE III.

John Foster, (colored,) a respectable man, aged twenty-nine years, suffering with phthisis pulmonalis, with emaciated frame, with profuse night sweats, and with general debility, accompanied with a troublesome cough, which prevented him from getting any rest, with a cavity in the right lung—no appetite, with great derangement of stomach and bowels. His condition when I was first called to see him, was of an extreme character, having little hopes of improvement or relief. I gave him "Firwein," along with cod liver oil—dose, tablespoonful three times a day, alternating with the compound syrups and *hypophosphites*, using counter irritation to skin, with croton oil, and gradually increasing the "Firwein," and giving him a good and nourishing diet. After using and keeping him under the influence of "Firwein" for some time, I found his condition somewhat changed, with decided improvement. There was decided amelioration of all the symptoms, a subsiding of the cough and profuse night sweats, a general building up of the whole system, and a degree of improvement in the lungs, which I have never seen before in all my professional experience with this fearful malady.

I believe that in this new compound, "Firwein," we have a desideratum long felt. I have never found a more potent remedial agent in arresting night sweats, and allaying the irritable condition of the digestive functions, usually so much impaired in "phthisis pulmonalis."

